# KatG, the Bifunctional Catalase of *Xanthomonas citri* subsp. citri, Responds to Hydrogen Peroxide and Contributes to Epiphytic Survival on Citrus Leaves

**DOI:** 10.1371/journal.pone.0151657

**Published:** 2016-03-18

**Authors:** María Laura Tondo, María Laura Delprato, Ivana Kraiselburd, María Verónica Fernández Zenoff, María Eugenia Farías, Elena G. Orellano

**Affiliations:** 1 Molecular Biology Division, Instituto de Biología Molecular y Celular de Rosario, Consejo Nacional de Investigaciones Científicas y Técnicas, Facultad de Ciencias Bioquímicas y Farmacéuticas, Universidad Nacional de Rosario, Rosario, Santa Fe, Argentina; 2 Planta Piloto de Procesos Industriales Microbiológicos, Consejo Nacional de Investigaciones Científicas y Técnicas, San Miguel de Tucumán, Tucumán, Argentina; East Carolina University School of Medicine, UNITED STATES

## Abstract

*Xanthomonas citri* subsp. citri (Xcc) is the bacterium responsible for citrus canker. This bacterium is exposed to reactive oxygen species (ROS) at different points during its life cycle, including those normally produced by aerobic respiration or upon exposition to ultraviolet (UV) radiation. Moreover, ROS are key components of the host immune response. Among enzymatic ROS-detoxifying mechanisms, catalases eliminate H_2_O_2_, avoiding the potential damage caused by this specie. Xcc genome includes four catalase genes. In this work, we studied the physiological role of KatG, the only bifunctional catalase of Xcc, through the construction and characterization of a modified strain (Xcc*katG*), carrying an insertional mutation in the *katG* gene. First, we evaluated the involvement of KatG in the bacterial adaptive response to H_2_O_2_. Xcc*katG* cultures exhibited lower catalase activity than those of the wild-type strain, and this activity was not induced upon treatment with sub-lethal doses of H_2_O_2_. Moreover, the KatG-deficient mutant exhibited decreased tolerance to H_2_O_2_ toxicity compared to wild-type cells and accumulated high intracellular levels of peroxides upon exposure to sub-lethal concentrations of H_2_O_2_. To further study the role of KatG in Xcc physiology, we evaluated bacterial survival upon exposure to UV-A or UV-B radiation. In both conditions, Xcc*katG* showed a high mortality in comparison to Xcc wild-type. Finally, we studied the development of bacterial biofilms. While structured biofilms were observed for the Xcc wild-type, the development of these structures was impaired for Xcc*katG*. Based on these results, we demonstrated that KatG is responsible for Xcc adaptive response to H_2_O_2_ and a key component of the bacterial response to oxidative stress. Moreover, this enzyme plays an important role during Xcc epiphytic survival, being essential for biofilm formation and UV resistance.

## Introduction

*Xanthomonas citri* subsp. citri (Xcc) is a Gram-negative, obligate aerobic, gamma proteobacterium responsible for citrus canker, a severe disease that affects most commercial citrus cultivars. The pathogen reaches its host by rain splash and wind and, after a period of epiphytic survival that can vary in length [1,2], it enters the host tissue through natural openings such as stomata and hydathodes or by way of wounds [3,4]. Subsequently, bacteria colonize the apoplast causing localized necrotic corky lesions on leaves, stems, and fruits. Disruption of the epidermis in the lesions allows the egress of bacteria to the surface and their dissemination to other tissues.

During its life cycle, Xcc is constantly exposed to reactive oxygen species (ROS), such us superoxide radical (O_2_^-^), hydrogen peroxide (H_2_O_2_) and hydroxyl radical (OH^.^), generated by consecutive univalent reductions of molecular oxygen [5]. ROS are produced inside bacterial cells, as a consequence of electron transport processes during aerobic respiration, and also by extracellular/environmental interactions.

Plants are usually in contact with different pathogens, and upon recognition ROS are rapidly produced at the site of infection. Their accumulation in high amounts in the apoplast is called “oxidative burst” and could be considered as a specific trait during the interaction with microorganisms [6–8]. ROS play multiple roles in plant defense; they act as signaling molecules in the plant immune response, prevent pathogen ingress through the oxidative cross-linkage of the plant cell wall, and impair bacterial growth at the site of attempted invasion [8]. Toxicity of ROS is based on their capacity to cause oxidative damage to all macromolecules, leading to DNA mutations, lipid peroxidation, disassembly of iron-sulfur clusters and other types of protein oxidation [9]. Thus, phytopathogenic bacteria must overcome an oxidative stress barrier in order to successfully colonize the host plant.

Other environmental factor disturbing ROS balance is ultraviolet radiation (UV-R). The incident light coming from the sun can be categorized, according to its wavelength, into UV-C (100–280 nm), UV-B (280–315 nm), and UV-A (315–400 nm). Only UV-B and UV-A are ecologically important as they reach the Earth’s surface even after a significant attenuation by ozone, cloud, aerosols, and water surface; UV-C is completely absorbed by the stratospheric ozone layer [10]. Bacteria are very susceptible to the effects of UV radiation, due to their small size, short generation time and absence of effective UV-protective pigmentation. A recent study aimed to identify the major determinants of bacterial inactivation under different UV wavelengths indicated that whereas the effects of UV-C radiation is entirely related to DNA damage, longer UV-A wavelengths have more indirect effects, producing the highest levels of intracellular ROS generation, lipid oxidation and protein carbonylation. The magnitude of UV-B effects was between those of UV-A and UV-C, supporting the suggestion that its action comprises elements from both pathways [11]. Plant leaf surfaces are normally exposed to high doses of incident UV radiation, as leaves are typically positioned for optimal exposure to sunlight for photosynthesis. Therefore, phyllosphere bacteria experiment the adverse biological effects of UV radiation, usually associated with oxidative stress.

Given the variety of conditions that causes ROS generation and in order to avoid toxicity, aerobic organisms have evolved a battery of enzymatic and non-enzymatic mechanisms dedicated to detoxify these species. Among them, catalases are devoted to eliminate H_2_O_2_, specie without net charge that can diffuse long distances inside the cell before reacting and causing massive damage. Although catalyzing the same reaction, the more than 300 catalase sequences now available can be classified into one of three groups according to their overall and active-site architecture and mechanism of reaction: (*i*) monofunctional heme-containing catalases, the most studied and widespread class; (*ii*) bifunctional heme-containing catalases, related to plant peroxidases both in sequence and structure; and (*iii*) nonheme or Mn-containing catalases [12,13].

The ability of Xcc to survive above and inside citrus leaves and fruits relies on its capacity to cope with ROS generation from multiple origins. In our previous work four putative catalase genes were identified *in silico* in the Xcc genome and their phase-regulated expression pattern were assayed by semi-quantitative RT-PCR, demonstrating that only three of them are effectively expressed. One of the monofunctional catalases, KatE, was shown to be the enzyme responsible for the increase in catalase activity during stationary phase and a main actor in Xcc colonization and survival in the apoplast of citrus plants [14]. Here we present a thorough study to the bifunctional catalase of Xcc, KatG. We show that this catalase is responsible for the adaptive response to H_2_O_2_ and a key component of the bacterial response to oxidative stress. Mutagenesis analysis also demonstrates that KatG is essential for biofilm formation and for UV resistance; actions required at the initial phase of host colonization, when bacteria must epiphytically survive on host leaves.

## Materials and Methods

### Bacterial strains, plasmids and growth conditions

Bacterial strains and plasmids used in this study are described in Table 1. Xcc strains were routinely grown aerobically in Silva Buddenhagen (SB) medium (5 g/L sucrose, 5 g/L yeast extract, 5 g/L peptone, and 1 g/L glutamic acid, pH 7.0) at 28°C with shaking at 200 rpm, or on 1.5% (w/v) Bacto agar-SB plates. *Escherichia coli* strains were grown at 37°C in Luria-Bertani (LB) medium [15]. Antibiotics were added to the media at the following final concentrations: ampicillin (Ap) 100 μg/mL for *E*. *coli* and 25 μg/mL for Xcc, kanamycin (Km) 40 μg/mL and gentamycin (Gm) 40 μg/mL for *E*. *coli* and 20 μg/mL for Xcc. Xcc strain Xcc99-1330 was kindly provided by Blanca I. Canteros (INTA Bella Vista, Argentina).

**Table 1:** Bacterial strains, plasmids and primers used in this work.

Strain/plasmid	Relevant genotype and description	Source/reference
**Strains**		
*Xanthomonas citri* subsp. citri		
Xcc99-1330	Wild-type, Ap^r^	B. I. Canteros
Xcc*katG*	*katG* mutant of Xcc99-1330, Km^r^, Ap^r^	This work
cXcc*katG*	Xcc*katG* complemented, carries pBBR1/*katG*, Km^r^, Gm^r^, Ap^r^	This work
*Escherichia coli*		
JM109	*HsdR17 endA1 Recal thi gyrA96 relA1 recA1 supE44*	[15]
	*λ*^-^*Δ(lac-proAB)*, [F’, traD36, proA^+^B^+^, *lacI*^*q*^*ZΔM15*]	
S17-1	*thi*, *pro*, *hsdR*, *recA* with RP4-2[Tc::Mu-Km::Tn7], Sm^r^	[16]
**Plasmids**		
pGEM-T Easy	PCR cloning and sequencing vector, Ap^r^	Promega
pGEM/*katG*	pGEM-T Easy containing 405-bp fragment of *katG*	This work
pK18mobGII	pUC18 derivative, *lacZa*, *gusA*, *mob* site, Km^r^	[17]
pKmob/*katG*	pK18mobGII containing 405-bp fragment of *katG*	This work
pBBR1MCS-5	Broad host-range vector, Gm^r^	[18]
pBBR1MCS-5EGFP	pBBR1MCS-5 containing *EGFP* gene	[19]
pBBR1/*katG*	pBBR1MCS-5 containing *katG* gene	This work
Primer name	Sequence^a^	Amplified fragment
katG-F1	5’ attggatccCTTGAAGACCTTCGGTTTTGC 3’	550 bp of the XCC1301 gene
katG-R1	5’ aattaagcttCAGATGGTCGAAGTAATCGTTG 3’	
katG-F2	5’ AGCATTCGAGCAAATCCAAC 3’	1241 bp of the XCC1301 gene
katG-R2	5’ GTCCTGCCACACCAACTCTTC 3’	
ckatG-F	5’ aattaagcttATCTGTTCGGCGACAAGG 3’	3032 bp including XCC1301
ckatG-R	5’ attggatccAGGTGGGTGTGAGTGAGGAC 3’	

Ap, ampicillin; Km, kanamycin; Gm, gentamycin; Sm, streptomycin.

^**a**^. Capital letters correspond to nucleotides of the Xcc genome sequence and small letters to nucleotides added to facilitate cloning.

### Recombinant DNA and microbiological techniques

All DNA manipulations including plasmid purification, restriction enzyme digestion, DNA ligation and agarose gel electrophoresis were performed with standard techniques [15]. Total bacterial genomic DNA from Xcc was isolated using the cetyltrimethylammonium bromide procedure [20]. Plasmids for bacterial conjugations were transferred to Xcc by biparental mating from the broad host-range-mobilizing *E*. *coli* strain S17-1 [16]. For this, bacterial mixtures were spotted onto Hybond-C membranes, placed on SB-agar and incubated for 48 h at 28°C. Membranes were then washed and bacteria transferred to selective medium as previously described [21].

### Construction of the Xcc*katG* mutant strain

The Xcc*katG* mutant was constructed by insertional inactivation of the chromosomal *katG* gene by a single homologous recombination. Primers katG-F1 and katG-R1 (Table 1) were used to amplify a 550-bp internal fragment of the *katG* coding region using Xcc genomic DNA as template. The PCR product was cloned into pGEM-T Easy vector (Promega), and the nucleotide sequence of the insert was confirmed by automated DNA sequencing. Subsequently, a *Hind*III-*Bam*HI fragment of the PCR product was subcloned into pK18mobGII [17], rendering the recombinant plasmid pKmob/*katG* (Table 1). This plasmid was then transferred from *E*. *coli* strain S17-1 [16] to the Xcc wild-type strain by conjugation. Recombination of the cloned *katG* fragment in the suicide plasmid with the homologous counterpart on the Xcc chromosome resulted in the disruption of the *katG* gene. The *katG* mutant was selected on SB-agar plates containing 40 μg/mL Km. *katG* inactivation was confirmed by PCR using the specific primers katG-F2 and katG-R2, located upstream and downstream of the gene fragment used for the homologous recombination (Table 1).

For mutant complementation, a 3032-bp DNA fragment containing the *katG* coding region and extending from 739 bp upstream of the 5’ end to 22 bp downstream of the 3’ end of the ORF was amplified using the primer pair ckatG-F and ckatG-R (Table 1). The amplified sequence included the putative promoter sequence of the *katG* gene, previously predicted with SoftBerry (www.softberry.com). The amplified DNA fragment was then cloned into the broad-host-range vector pBBR1MCS-5 [18] to generate the recombinant plasmid pBBR1/*katG*. This plasmid was transferred into the Xcc*katG* mutant strain by conjugation, rendering strain cXcc*katG* (Table 1).

### Enzyme activity assay and staining

Cell extracts were prepared from 10 mL cultures harvested by centrifugation at 10000 *g* for 10 min at 4°C. Bacteria were washed and resuspended in 500 μL of ice-cold 50 mM potassium phosphate buffer (pH 7.0) containing 1 mM PMSF, and then disrupted by intermittent sonication. Suspensions were clarified by centrifugation at 12000 *g* for 20 min at 4°C. Protein concentrations in soluble cell extracts were determined by the Sedmak and Grossberg method [22], with bovine serum albumin as standard.

Catalase activity in cell extracts was monitored through the decomposition of hydrogen peroxide by following the decrease in absorbance at 240 nm [23]. The assays were performed at 25°C in 50 mM potassium phosphate buffer (pH 7.0), containing 10 mM H_2_O_2_. An extinction coefficient of 43.6 M^-1^ cm^-1^ at 240 nm was used to calculate the specific activity. One unit of catalase activity was defined as the amount of activity required to decompose 1 μmol of H_2_O_2_ per minute under the assay conditions.

For evaluation of catalase activity in gels, aliquots of cell extracts containing 25–50 μg of soluble protein were electrophoresed on 8% (w/v) non-denaturing polyacrylamide gels and stained for catalase activity as described by Scandalios [24]. To eliminate the likelihood of multiple, potentially artifactual catalase bands, which can be detected at higher amperage (20 to 30 mA), non-denaturing gels were electrophoresed at 10 mA to resolve those bands.

Peroxidase activity staining was performed according to Kang *et al*. [25] with some modifications. Briefly, the cell extracts (25 μg) were electrophoresed on 8% (w/v) native polyacrylamide gels, after which gels were incubated in 0.1 M Tris-HCl (pH 7.5) containing 0.1 mg/mL 3,3’-diaminobenzidine, 9 mM H_2_O_2_ and 0.4 mg/mL NiCl_2_ for approximately 20 min in the dark, until appearance of the bands.

### Survival in the presence of hydrogen peroxide

For the induction experiments, Xcc cultures were grown to early exponential phase (5 h) and incubated with sub-lethal concentrations of hydrogen peroxide (10, 30 and 100 μM) for an additional hour before being used in the killing experiments. After the induction treatment, aliquots of the cultures were washed, diluted and plated on SB-agar plates. Cultures were then treated with a lethal concentration of H_2_O_2_ (1 mM) for 15 min, after which samples were taken, washed once with fresh medium, serially diluted and plated on SB-agar plates.

To test bacterial resistance to 100 μM H_2_O_2_, early exponential phase cultures were diluted and plated on SB-agar plates prior to addition of the oxidant. After 1 hour of incubation with 100 μM H_2_O_2_, samples were removed, washed once with fresh medium, serially diluted and plated on SB-agar plates. In all cases, growth of liquid cultures was monitored spectrophotometrically by optical density at 600 nm (OD_600_). Colonies were counted after 48 h incubation at 28°C. The percentage of survival was defined as the number of colony forming units (CFU) after treatment divided by the number of CFU prior to treatment ×100.

### UV resistance assays

To evaluate UV-A and UV-B resistance, Xcc strains were grown in SB medium at 28°C and 200 rpm to early exponential phase (5 h). Cells were harvested by centrifugation at 10000 *g* for 15 min at 4°C, and the pellets were washed and resuspended in 0.9% (w/v) NaCl. Aliquots of each cell suspension were transferred to 45-mL sterile quartz tubes and exposed to UV-A (Lamp 09815–00, Cole-Parmer Instruments Company, average intensity: 2.67 W m^-2^) or UV-B (Lamp 09815–06, Cole-Parmer Instruments Company, maximum intensity at 312 nm, average intensity: 2.5 W m^-2^) radiation with slow shaking (25 rpm) at 20°C. UV-A and UV-B doses were adjusted by varying the exposure times. Controls were incubated in the dark under the same conditions. After UV exposure, 100 μL-aliquots were removed from the tubes and microbial growth was assessed by counting the number of CFU after 48 h of incubation at 28°C in the dark to prevent photoreactivation. Experiments were performed in triplicate. A two-factor (UV dose and strain) mixed model ANOVA and a Tukey’s multiple comparison test were used for statistical analyses. Residual analyses and validation with logarithmic data transformation were performed.

### ROS (-OOH) detection

A modified version of the FOX II assay [26] was used to quantify the presence of peroxides in bacterial protein extracts. Cultures of the parental Xcc and the *katG* mutant were grown in SB medium at 28°C to early exponential phase (5 h) with the appropriate antibiotics. Then, H_2_O_2_ was added to a final concentration of 100 μM, and bacterial suspensions were incubated for 1 hour with vigorous shaking. Cultures were split into two equal portions; one of them was used to measure protein concentration, while the other was centrifuged, washed with 0.9% (w/v) NaCl and finally resuspended in 1 mL of an 80:20 ethanol/water solution containing 0.01% (w/v) butylated hydroxytoluene (BHT). Samples were disrupted by intermittent sonication (20 times for 10 sec each, 30% amplitude), centrifuged at 10,000 *g* for 10 min, and 250 μL of the supernatants were combined with 250 μL of 10 mM Tris-phenyl phosphine in methanol (TPP, a -OOH reducing agent), or with 250 μL of methanol, to measure total oxidants. Mixtures were incubated for 30 min to allow complete -OOH reduction by TPP. Then, 500 μL of FOX reagent (100 μM xylenol orange, 4 mM BHT, 250 μM ferrous ammonium sulphate and 25 mM H_2_SO_4_ in 90% (v/v) methanol) were added to each sample, and the absorbance at 560 nm was recorded exactly 10 min after reagent addition. The absorbance differences between equivalent samples with and without TPP indicate the amounts of -OOH, calculated according to a 0–20 μM H_2_O_2_ standard curve. Protein concentrations were estimated in cleared lysates by a dye binding method [22], using bovine serum albumin as standard.

### Biofilm formation assays

Xcc strains were modified to express the green fluorescence protein (GFP). Briefly, the coding sequence for EGFP from pEGFP-1 (Clontech, Palo Alto, CA, U.S.A.) was digested with *Bam*HI and *Xba*I and ligated in-frame with the LacZ-α-peptide of the broad-host-range vector pBBR1MCS-5 [18] previously digested with the same enzymes, rendering the plasmid pBBR1MCS-5EGFP [19]. *E*. *coli* S17-1 cells harbouring this plasmid were conjugated to Xcc strains, and transconjugants were selected for Gm resistance. Saturated cultures of the GFP-labeled bacteria in SB medium were adjusted to the same OD_600_ and diluted 1:100 in fresh medium, and 300 μL was placed onto chamber-covered glass slides (nu155411, Lab-Tek, NUNC, Naperville. IL, U.S.A.). Chambers were statically incubated in a humidified PVC-box at 28°C. Biofilm formation was visualized by confocal laser scanning microscopy (Nikon Eclipse TE-2000-E2) with a motor system and DIC/Nomarski optics and a head scan D Eclipse C1si. The images obtained were analyzed with Nikon EZC1 3.90 software.

Biofilm formation was also studied on glass tubes. Saturated cultures of Xcc in SB medium were adjusted to OD_600_ = 1. Subsequently, 20 μL of each bacterial culture were transferred to glass tubes containing 2 mL of fresh medium and statically incubated at 28°C. After 12 days, cultures were poured out and tubes were rinsed twice with distilled water. Following fixation at 60°C for 20 min, biofilms on the glass surface were stained with 0.1% (w/v) crystal violet for 45 min. Tubes were then washed 3 times with distilled water and quantitatively analyzed by solubilising the stained biofilms with a solution of 10% (v/v) acetic acid and 90% (v/v) ethanol for 2 h, and then measuring absorbance at 540 nm of the stained suspension in a spectrophotometer.

### Plant material and plant inoculations

Orange (*Citrus sinensis* cv. Valencia) was used as the host plant for Xcc. Plants were grown in a growth chamber under incandescent light, at 25°C and a photoperiod of 16 h. Overnight cultures of Xcc WT, Xcc*katG* and cXcc*katG* were diluted in 10 mM MgCl_2_ to a final concentration of 10^5^ CFU/mL. For disease symptoms assays, bacterial suspensions were infiltrated into leaves with needleless syringes. *In planta* growth assays were performed by grinding 0.8 cm diameter leaf discs from infiltrated leaves in 100 μL of 10 mM MgCl_2_, followed by serial dilutions and plating onto SB-agar plates. Colonies were counted after 48 h incubation at 28°C.

For epiphytic fitness assays, overnight bacterial cultures were adjusted to 10^8^ CFU/mL in 10 mM potassium phosphate buffer (pH 7.0). Five leaves of three independent orange plants were inoculated with the Xcc WT, Xcc*katG* and cXcc*katG* strains by placing eight 5-μL drops of each bacterial suspension in a defined circular area (1.5 cm^2^) of the leaf; therefore, each leaf supported a total volume of 40 μL of each bacterial suspension. After absorption of the drops, leaves were covered with wet plastic bags for 24 h to maintain high humidity. At each time point, disks of 1.5 cm^2^ corresponding to the different inoculated areas of every leaf, were cut and transferred to 0.4 mL of 10 mM potassium phosphate buffer (pH 7.0). Tubes were submerged in a Branson model #5510 sonicator for 10 min. Subsequently, leaf disks were stirred gently for 1 min and serial dilutions were plated on SB-agar plates containing appropriate antibiotics for each strain.

### Statistical analysis

Quantitative analyses were performed with at least three independent biological samples. Data were subjected to a multifactorial mixed model ANOVA and Tukey’s multiple comparison tests along with residual analysis and validation using Infostat software (Infostat 2006H, http://www.infostat.com.ar).

## Results and Discussion

### KatG is induced during the adaptive response of Xcc to hydrogen peroxide

We have previously shown that upon exposure to sub-lethal concentrations of H_2_O_2_ Xcc induces catalase activity and this response enhances bacterial ability to withstand a lethal dose of the same oxidative agent [14]. In many bacterial species this “adaptive response” to H_2_O_2_ occurs as a result of transcriptional induction of an specific catalase gene [27,28]. In *E*. *coli* and *Salmonella typhimurium*, the levels of the catalase-peroxidase KatG increases in response to added H_2_O_2_ in a reaction mediated by OxyR [29].

**Fig 1:**
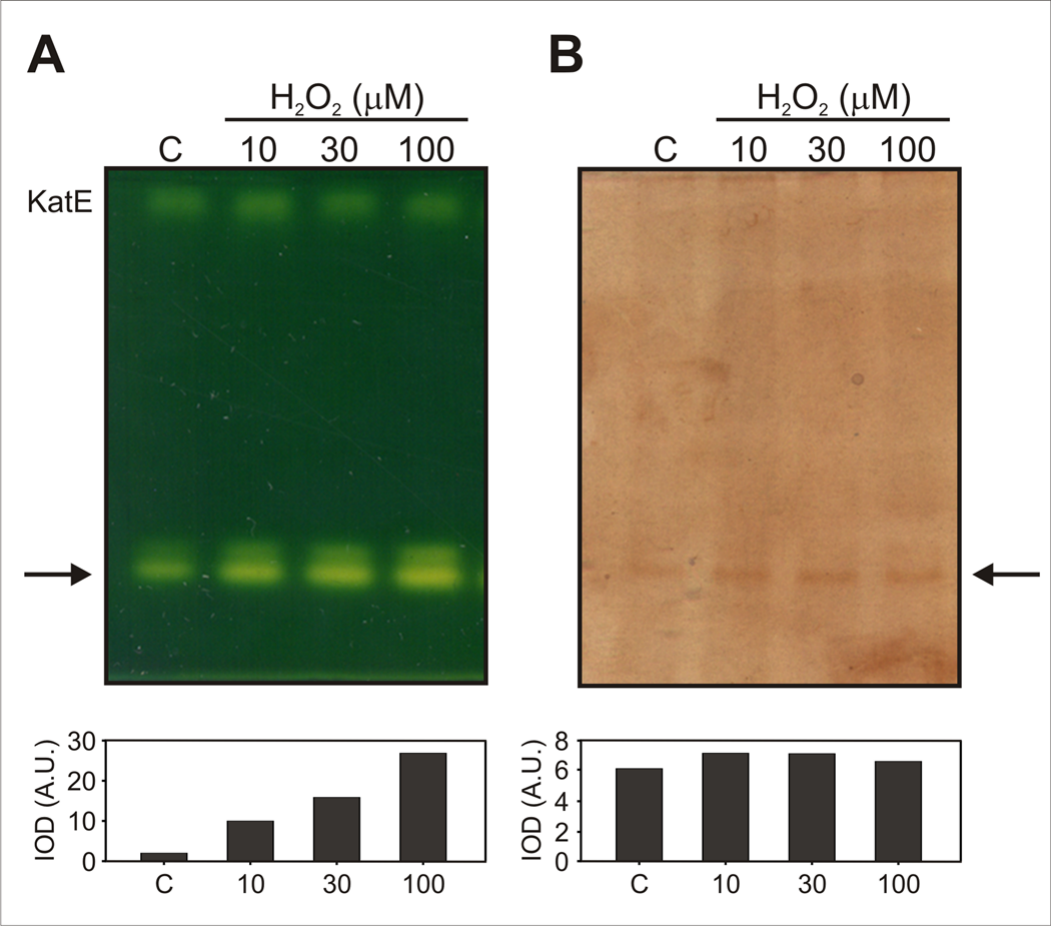
Detection of catalase and peroxidase activities in Xcc cultures adapted with hydrogen peroxide. Exponential phase cultures were treated with the indicated concentrations of H_2_O_2_ for 60 min, and soluble extracts were prepared as described in the experimental section. Equal amounts of protein (25 μg) were separated in duplicate on 8% non-denaturing polyacrilamide gels stained for catalase (A) and peroxidase (B) activities. The position of the single catalase-peroxidase species detected is indicated by an arrow. Histograms below gels show the activity profiles obtained by densitometric quantification of the fast-migrating bands intensities. IOD, integrated optical density; A.U., arbitrary units. C, untreated control culture.

When the effect of sub-lethal levels of H_2_O_2_ (10–500 μM) on catalase gene expression was analyzed by semi-quantitative RT-PCR in Xcc, no induction of any of the genes was observed, indicating that the previously reported H_2_O_2_-mediated catalase induction [14] was not regulated at the transcriptional level (data not shown). As an alternative approach to assess whether KatG was the catalase responsible for the adaptive response of Xcc to H_2_O_2_, equal amounts of soluble protein extracts from cultures pre-adapted with 10, 30 and 100 μM H_2_O_2_ and from an untreated control were separated in duplicate on a non-denaturing polyacrylamide gel; one half of the gel was stained for catalase activity and the other one was stained with the donor 3,3’-diaminobenzidine for peroxidase activity. As shown in Fig 1, the band with the highest electrophoretic mobility was found to exhibit also peroxidase activity (Fig 1B), suggesting that it corresponds to KatG, the only bifunctional catalase-peroxidase identified in the Xcc genome sequence [30,31]. Gel densitometric analysis indicated that the catalase activity of this band increased gradually with the H_2_O_2_ concentrations used for the treatments, reaching a ~13-fold induction upon adaptation with 100 μM H_2_O_2_, and strongly suggesting that KatG may be involved in the adaptive response of Xcc to H_2_O_2_. Regarding the apparently absence of induction of the peroxidase activity, recent reports demonstrated that in bifunctional catalases, structural changes in the protein can differentially affect the catalase and peroxidase activities, i.e., alterations in specific amino acidic residues can impair the catalase activity while having small effects on the peroxidase activity [32]. This observation suggests that in these proteins, catalase and peroxidase functions can be independently regulated. In this way, it would be plausible for both activities to be differentially influenced by environmental conditions that affect the protein structure.

**Fig 2:**
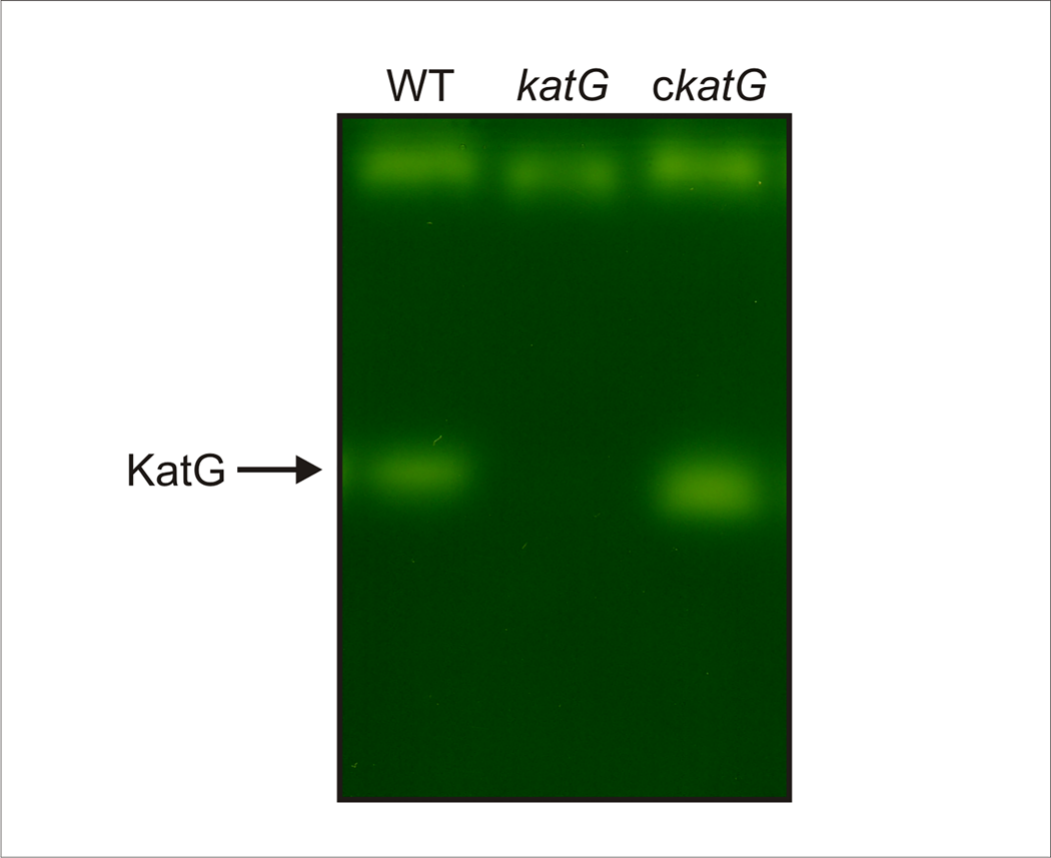
Catalase activity pattern in the Xcc*katG* mutant. Xcc wild-type (WT), Xcc*katG* (*katG*) and cXcc*katG* (*ckatG*) strains were grown aerobically in SB medium to early exponential phase (4 h), and soluble extracts were prepared as described in the experimental section. Equal amounts of protein (25 μg) were separated by 8% (w/v) non-denaturing PAGE and stained for catalase activity [24].

In order to confirm the identity of the fast-migrating band and to evaluate the role of KatG in Xcc physiology a mutant strain, Xcc*katG*, was constructed by insertional mutagenesis and genetically verified by PCR analysis (data not shown). Soluble protein extracts from the parental (WT), and mutant (*katG*) strains in early exponential phase of growth were then analyzed by native gel electrophoresis and catalase staining. As shown in Fig 2, the apparently two fast migrating bands observed in wild-type cells were completely absent in the *katG* mutant, indicating that both bands derive from the same gene locus. The presence of two bands for KatG in the gel presented in Fig 1 could be the consequence of slight conformational differences in the protein structure that affect the electrophoretic mobility in native gels, or be an artificial effect of the electrophoretic run. However, the possibility of a post-translational modification of the KatG protein cannot be ruled out even though this type of regulation has not been reported in bacterial catalases.

To validate this result, a complementation assay was carried out by cloning the *katG* gene under the control of its own promoter sequence in a pBBR1-MCS5 vector [18], which was then conjugated into the Xcc*katG* mutant strain. The complemented cXcc*katG* strain recovered the catalase pattern observed for Xcc wild-type, corroborating the identity of this band (Fig 2).

Thus, our results clearly suggest that Xcc exposure to sub-lethal doses of hydrogen peroxide leads to an increase in KatG activity, without detectable changes in *katG* transcript levels. In this regard, it has recently been suggested that the activity of bifunctional KatG may be affected in a translational or a post-translational step in a *Caulobacter crescentus rho* mutant, based on the observation that catalase activity is drastically decreased in this strain even when the transcript levels of its unique catalase gene are higher than in wild-type cells [33]. However, the nature of this regulatory mechanism is still unknown.

### KatG is responsible for the adaptive response to hydrogen peroxide and essential for the resistance to oxidative stress

The ability of Xcc*katG* to develop an adaptive response to H_2_O_2_ was then evaluated by measuring total catalase activity in early exponential cultures incubated with sub-lethal concentrations of H_2_O_2_ (10, 30 and 100 μM) for 60 min (Table 2). As previously reported [14], Xcc wild-type exhibited a dose-dependent response to these treatments, reaching a 2-fold induction of catalase activity when exposed to 100 μM H_2_O_2_. In contrast, no induction was observed in the Xcc*katG*-treated cultures, being the basal catalase level of this strain (uninduced culture) considerably lower than that of the wild-type cells (~2.5-fold).

**Table 2:** Catalase activity of Xcc cultures in response to sub-lethal levels of hydrogen peroxide^a^.

	Xcc WT		Xcc*katG*	
	Catalase activity (μmol min^-1^ mg^-1^ protein)	Induction (fold)	Catalase activity (μmol min^-1^ mg^-1^ protein)	Induction (fold)
Uninduced	4.3 ± 0.1	-	1.7 ± 0.1	-
Induced by H_2_O_2_				
10 μM	4.7 ± 0.2	1.1	1.6 ± 0.2	0.9
30 μM	6.9 ± 0.1	1.6	1.7 ± 0.1	1.0
100 μM	9.1 ± 0.3	2.1	1.5 ± 0.3	0.8

^**a**^. Xcc cells were grown in SB medium to early exponential phase and exposed to the indicated concentrations of H_2_O_2_ for 1 hour. Catalase activities in soluble cell extracts were measured as described in Materials and Methods.

Data represent mean ± standard deviation of three independent experiments.

**Fig 3:**
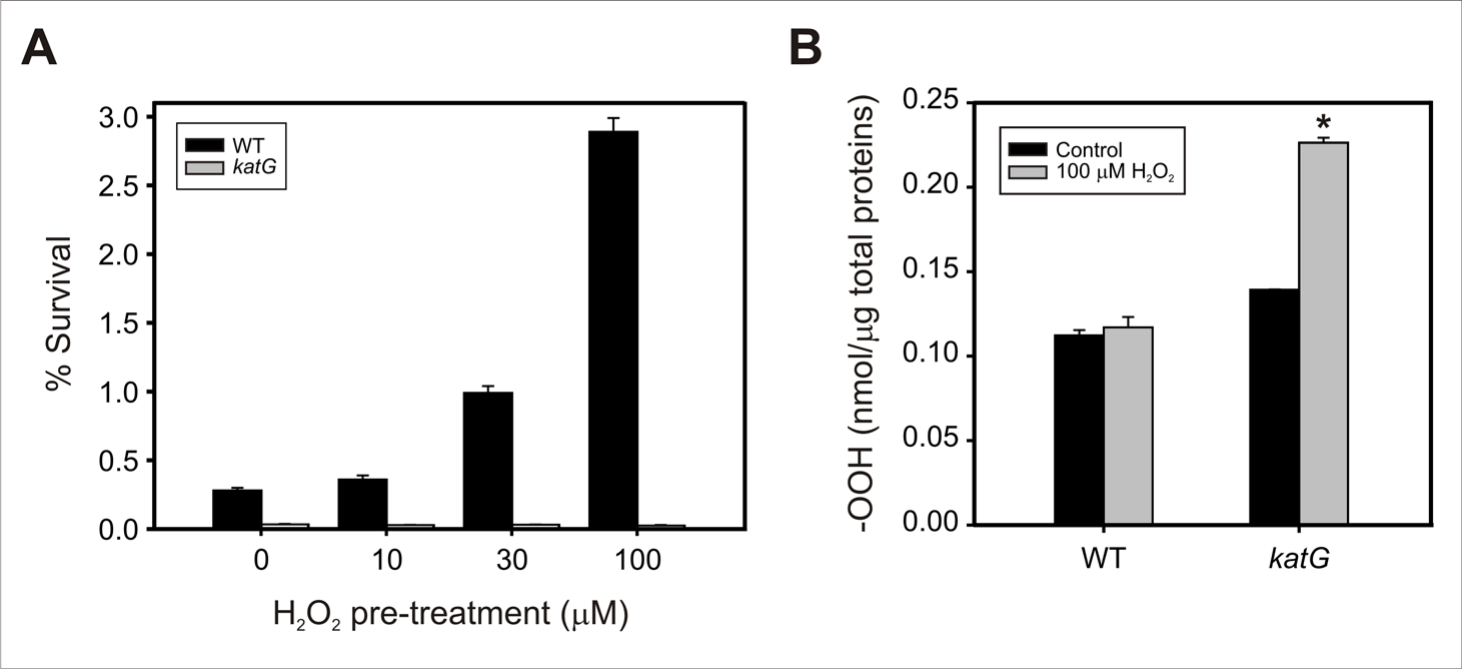
Sensitivity of Xcc*katG* to hydrogen peroxide. (A) Hydrogen peroxide resistance of pre-adapted Xcc cells. Exponential phase cultures of Xcc wild-type and *katG* mutant were adapted with the indicated concentrations of H_2_O_2_ for 60 min and then exposed to 1 mM H_2_O_2_ for 15 min. The number of CFU was determined for each culture before and after the treatment with 1 mM H_2_O_2_ by plating of appropriate dilutions. The percentage of survival was calculated as the number of CFU after treatment divided by the number of CFU prior to treatment ×100. Data represent mean ± standard deviation of three independent experiments. (B) ROS accumulation upon exposure to hydrogen peroxide. Bacteria were exposed to 100 μM H_2_O_2_ for 1 hour, and total peroxides (-OOH) were determined in cleared extracts using the FOX II assay as described in the experimental section. Measurements were carried out in triplicate for two independent experiments, and the results are expressed as means ± standard deviations. Statistical significant differences (P < 0.05, ANOVA) between wild-type and *katG* strains are indicated by an asterisk.

Resistance of bacterial cells pre-adapted with sub-lethal levels of H_2_O_2_ to a lethal dose of the same agent (1 mM) was then assessed (Fig 3A). Survival of Xcc wild-type clearly increased with the adaptive treatments and the level of induced protection correlated with the H_2_O_2_ concentration used for the adaptation, as previously demonstrated [14]. Conversely, the *katG* mutant was found to be extremely sensitive to 1 mM H_2_O_2_ (~8-fold more sensitive than the parental strain in control conditions) and did not exhibit increments in the percentage of survival after pre-adaptation with low concentrations of the oxidant.

Based on these observations we further tested whether the pre-adaptation treatment may result harmful to Xcc*katG*, presumably the cause of the absence of an adaptive response to H_2_O_2_. For that purpose, ROS accumulation in bacterial cell extracts from the wild-type and *katG* strains after exposure to 100 μM H_2_O_2_ for 1 hour (adaptive treatment) was quantified. As shown in Fig 3B, ROS (-OOH) levels did not increase in wild-type cells when treated with this concentration of the oxidant. In contrast, a significant ROS build-up was observed in mutant cells under this condition, with a rise of 1.6-fold relative to the untreated control culture. An increase of similar magnitude in the intracellular ROS (-OOH) levels was determined for a flavodoxin mutant of *Pseudomonas aeruginosa* when challenged with a significantly higher dose of H_2_O_2_ (25 mM), associated with a decreased tolerance to H_2_O_2_ toxicity compared to wild-type cells [34]. We also determined bacterial viability of Xcc wild-type and *katG* after challenge with 100 μM H_2_O_2_ for 1 hour. Cells of the wild-type strain displayed complete resistance to this treatment (100% survival) as this H_2_O_2_ concentration was previously shown to be a sub-lethal dose for Xcc [14]. In contrast, only ~20% survival was determined for the *katG*-deficient mutant strain, denoting a remarkably high sensitivity to H_2_O_2_ (data not shown). Thus, our results collectively indicate that exposure of the *katG* mutant to this very low dose of hydrogen peroxide leads to an intracellular ROS (-OOH) build-up that compromise bacterial viability.

The high sensitivity of Xcc*katG* to oxidative stress may also explain the slower growth kinetic of this strain in liquid medium (S1 Fig), which suggests that it has an impaired ability to deal with ROS endogenously generated as a consequence of aerobic respiration. In agreement, bifunctional catalases KatG from *Pseudomonas syringae* pv. tomato [35] and KatA from *Agrobacterium tumefaciens* [36] were found to be involved in the removal of endogenous H_2_O_2_, intracellularly generated through menadione reduction.

Many bacterial species contain catalase genes paralogues in their genomes, being one enzyme specifically induced by oxidative stress conditions [13]. Based on the existing information regarding the physiology of catalase expression and its control in bacteria, Mishra and Imlay have observed that bifunctional catalases are usually the preferred enzymes during exponential growth and the ones induced in the presence of environmental H_2_O_2_ by the action of the OxyR or PerR systems. In contrast, monofunctional catalases are commonly induced upon entry into stationary phase by specific sigma factors [37]. In agreement, we have previously observed that KatG is the main catalase expressed in Xcc during the exponential phase of growth [14]. The results obtained in this work, particularly the induction of KatG band observed in native gels after H_2_O_2_ treatment (Fig 1), the absence of an adaptive response to H_2_O_2_ in Xcc*katG* mutant and the extremely high sensitivity to the oxidant exhibited by this strain, allowed us to postulate KatG as the catalase specifically involved in the response of Xcc to environmental H_2_O_2_.

Participation of the catalase-peroxidase isozyme in the protection against micromolar concentrations of H_2_O_2_ has also been reported in *Xanthomonas campestris* pv. campestris, as part of the set of genes induced by the OxyR regulon [38]. Noticeably, RT-PCR analysis aimed to detect the transcriptional induction of catalase genes in Xcc cultures challenged with H_2_O_2_ in a 10–500 μM concentration range revealed no differences in the transcript levels of *katG* or any other catalase gene (data not shown). Therefore, the increase in KatG activity observed in Xcc under these conditions would be most likely the result of a post-transcriptional regulation, in contrast to that observed in other *Xanthomonas* species. Other studies would be needed to probe this contention.

### KatG plays a significant role in the Xcc resistance to UV radiation

**Fig 4:**
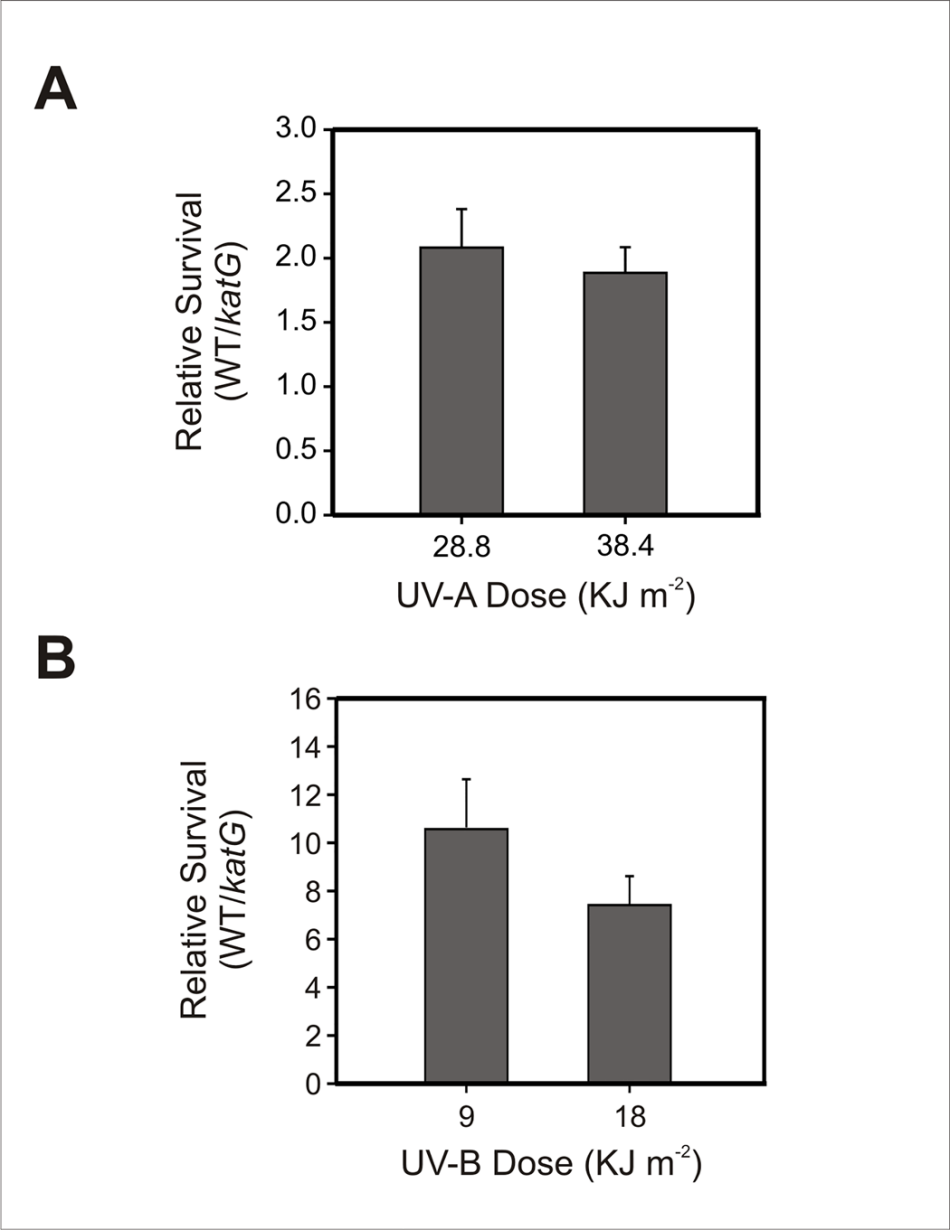
Sensitivity of Xcc*katG* to UV radiation. Exponential phase cultures of Xcc wild-type and *katG* mutant were exposed to the indicated doses of UV-A (A) and UV-B (B) radiation and the number of CFU after treatment was determined for each culture by plating of appropriate dilutions. The percentages of survival were calculated in relation to control cultures that were incubated in the dark under the same conditions. Relative survival is defined as the percentage of survival of the wild-type strain divided by the percentage of survival of the *katG* mutant strain. Experiments were performed in triplicate; values are expressed as means ± standard deviations. Statistical significant differences (P < 0.05, ANOVA) between wild-type and *katG* strains were observed in all treatments.

To further evaluate the role of KatG in Xcc physiology, survival of the *katG* deficient mutant following exposure to either UV-A or UV-B radiation was compared to that of its parental wild-type strain. We found that the absence of KatG in the mutant strain produced an approximately 2-fold decrease in survival after exposure to UV-A doses (Fig 4A) and a >7-fold decrease in survival after exposure to UV-B (Fig 4B). These results suggest that catalase KatG plays a role in Xcc defense response against wavelengths of UV light encountered in natural solar radiation.

Several lines of evidence indicate that both UV-A and UV-B wavelengths lead to oxidative stress in bacteria through enhanced production of ROS, and this explain why many bacterial species induce the expression of antioxidant enzymes following UV irradiation [39–41]. Unusually high catalase activity was observed in strongly UV-tolerant *Acinetobacter* isolates, and it was concluded that this enzyme plays a role in the response to UV radiation [42]. On the other hand, a strong correlation between catalase levels and UV-A sensitivity was observed in *P*. *aeruginosa* PAO1, demonstrating the important role of catalase activity against UV-A induced damage [43]. In addition, a catalase-deficient *katA* derivative of PAO1 was found to be more sensitive to UV-A radiation than the wild-type strain under both planktonic and biofilm growth [44]. The protective role of catalases against UV damage may possibly be related to the rapid removal of hydrogen peroxide generated by the radiation, before highly toxic hydroxyl radical could be produced by the Fenton and Haber-Weiss reactions. It has also long been reported that bovine catalase absorbs light of UV-A wavelengths with its consequent inactivation [45,46]. This observation led to the proposal that catalases might also act by preventing radiation from reaching photosensitizer molecules, thus diminishing the production of ROS [44]. Whatever the case, catalases have proven to be important components of the bacterial defense response against UV radiation, and their contribution might be particularly significant for phytopathogens like Xcc that must survive on sunlight-exposed leaf surfaces before colonization.

### KatG disruption affects biofilm formation

Biofilms constitute a microbial multicellular lifestyle wherein groups of microcolonies are attached to an inert or living surface and enclosed in a self-produced polymeric matrix mainly composed of exopolysaccharide [47,48]. It has been previously demonstrated that Xcc forms biofilms on both biotic and abiotic surfaces, and this mode of growth has an important role for the epiphytic survival and disease development in host plants [2,49,50].

**Fig 5:**
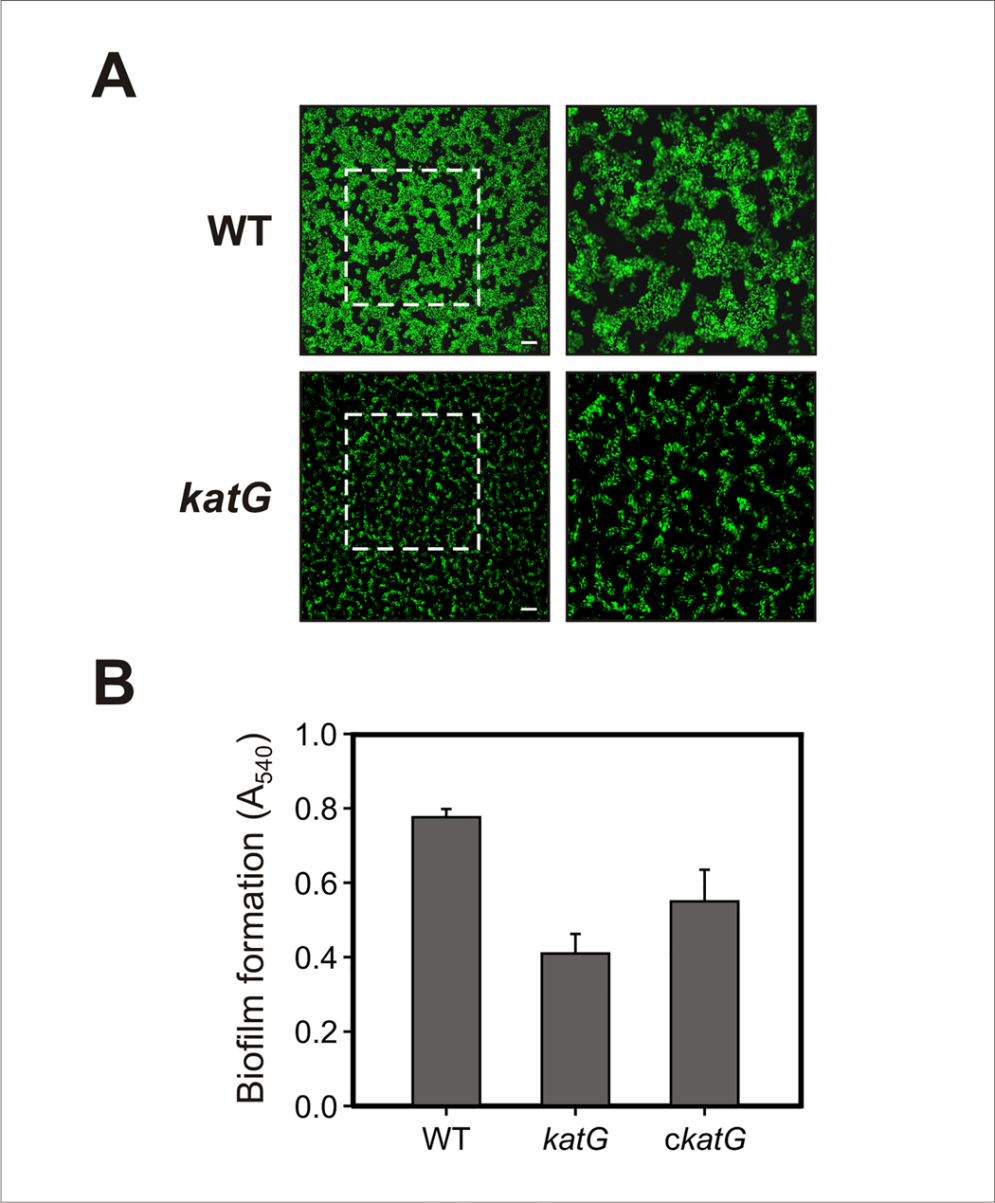
Effect of *katG* disruption on biofilm formation. (A) GFP-labeled Xcc strains were grown on chambered cover slides and visualized under confocal laser scanning microscopy (CLSM) after 2 days of bacterial growth. Left panels show the biofilms developed at the bottom of the chambered cover slides with a magnification of 400X and right panels show a 2X zoom of the regions marked in the previous panels. Scale bars, 50 μm. (B) Xcc strains were statically grown on glass tubes for 12 days at 28°C. Biofilm formation levels on the air-liquid interface were determined by crystal violet staining. The results show the means and standard deviations of a representative experiment with triplicate samples. The experiment was repeated three times with similar results in all cases.

To investigate a possible role of catalase KatG in the biofilm formation of Xcc, we used confocal laser scanning microscopy (CLSM) to analyze the morphology of bacterial biofilms developed by green fluorescent protein (GFP)-labeled strains of Xcc on chambered cover glass slides. After two days of static incubation Xcc wild-type developed a structured biofilm in which bacteria were densely packed and organized forming large aggregates that extended over the entire surface. In contrast, *katG* mutant cells generated smaller disperse microcolonies that were considerably less organized than those produced by Xcc wild-type (Fig 5A). We also assayed the ability of Xcc to develop a biofilm on glass tubes containing SB liquid medium, in order to quantitatively measure the biofilm formation level of the strains after longer incubation times. After 12 days of static incubation at 28°C we observed cell aggregates on the air-liquid interface that were subjected to crystal violet staining. As shown in Fig 5B, the *katG* mutant displayed a significant decrease in the level of biofilm formation compared with the wild-type strain, confirming our previous observations; on the other hand, complementation of the *katG* mutant only partially restored biofilm formation to the wild-type level. These results suggest that the *katG* gene, involved in the Xcc oxidative stress response, contributes to the development of a mature biofilm.

Biofilm development is known to be influenced by genetic and environmental factors and involves deep changes in bacterial gene expression. Among the proteins that exhibit enhanced expression in biofilms compared with planktonic (free-living) cells, several proteins involved in the resistance to oxidative damage have been reported [51–53], including superoxide dismutase (SodC) and thioredoxin-dependent thiol peroxidase (Tpx) in *E*. *coli* O157:H7 [54] and catalase (KatA) in *P*. *aeruginosa* [55]. The aggregated bacterial growth within biofilms limits the penetration of nutrients and oxygen as well as the diffusion of metabolic waste products, leading to a variety of nutritional and physiological stresses in biofilm cells [48]. The increased expression of antioxidant systems in biofilms gives a clear indication that cells may also experience oxidative stress under this growth condition. In fact, endogenous oxidative stress has been shown to increase mutation frequencies producing genetic diversity and antibiotic resistance in biofilm cells [56,57]. Therefore, the requirement of KatG for the establishment of a mature biofilm in Xcc supports the notion that endogenous ROS generation affect bacterial physiology in biofilm-growing cells and antioxidant systems may serve a key role to alleviate this stress during biofilm development. In agreement, *sodC* and *tpx* mutants of *E*. *coli* O157:H7 have been shown to exhibit an impaired ability to form biofilms on both biotic and abiotic surfaces [54].

### Interaction of the Xcc*katG* mutant with orange plants

**Fig 6:**
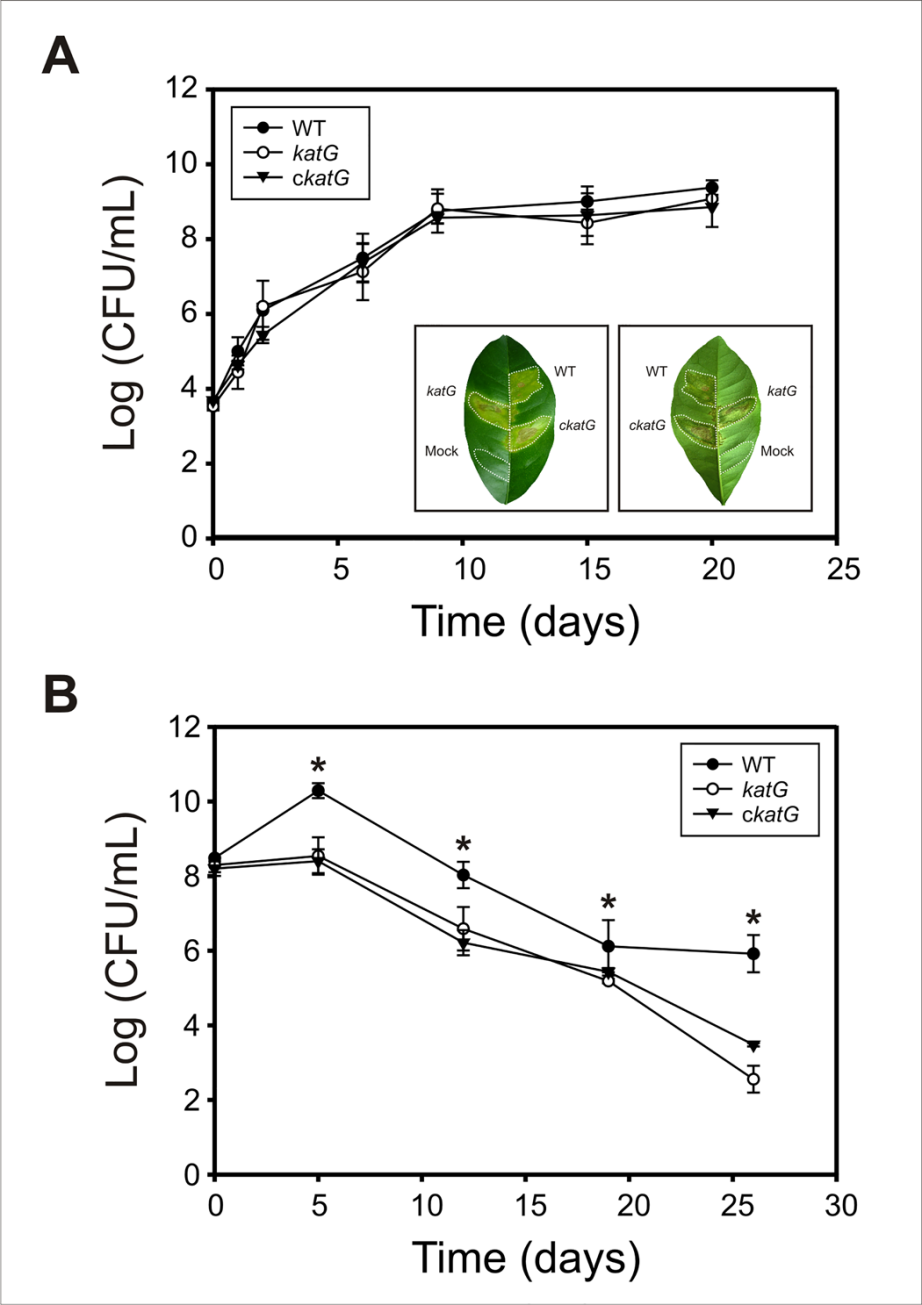
Pathogenicity and epiphytic fitness of Xcc*katG* in orange plants. (A) Growth of Xcc strains in the apoplastic space of orange leaves. Xcc WT, Xcc*katG* and cXcc*katG* cells were inoculated at 10^5^ CFU/mL in 10 mM MgCl_2_ into the intercellular spaces of fully expanded orange leaves. Bacterial populations in leaf tissues were determined by serial dilution and plating. A representative leaf 20 days after inoculation with the three strains is shown in the lower inset. Left panel, adaxial side; right panel, abaxial side. Dashed lines indicate the infiltrated area. (B) Epiphytic populations of Xcc strains on orange leaves. Bacterial cells were released from the leaf surface by sonication followed by dilution plating. Experiments were performed in triplicate; values are expressed as means ± standard deviations. Statistical significant differences (P < 0.05, ANOVA) between wild-type and *katG* strains are indicated by an asterisk.

In order to assess whether *katG* mutation affects Xcc virulence, *Citrus sinensis* (orange) leaves were inoculated with cultures of Xcc wild-type, *katG* mutant and complemented c*katG* strains adjusted to 10^5^ CFU/mL. Phenotypic observation of the infiltrated leaves showed no differences in the development of canker lesions, which were similar in magnitude, number and time of appearance for the three Xcc strains (Fig 6A). We also determined the bacterial populations *in planta* at different times after inoculation, observing that the mutant strain was able to multiply inside plant tissues with similar growth kinetics and to the same extent as wild-type cells (Fig 6A).

The adaptive response to oxidative agents has been proposed to serve a protective role against increased oxidative stress conditions during bacterial interactions with plants [58]. Even though KatG was shown to be responsible for the Xcc adaptive response to hydrogen peroxide, the major component of the plant oxidative burst [59,60], our results indicate that the deficiency in this enzyme does not affect Xcc survival and establishment in the apoplast of orange leaves. Consistent with these findings, we have previously demonstrated that the KatE isozyme is specifically induced in the apoplast environment, leading to an increase in total catalase activity and in the bacterial resistance to H_2_O_2_. In fact, the Xcc *katE*-deficient mutant was found to exhibit an impaired ability to grow in host tissues and to cause disease, indicating that KatE plays a major role in the bacterial battle against plant-produced ROS [14]. The induction of KatE inside host tissues may also explain the fact that Xcc*katG* does not exhibit growth restrictions *in planta* even though it displays reduced growth rates during *in vitro* culture in SB medium (S1 Fig).

Generation of ROS in the apoplast of host tissues is not the only source of oxidative stress to which Xcc is exposed during the interaction with plants and the repertoire of Xcc catalases might have evolved to protect cells in each stage of the bacterial life cycle. Thus, we hypothesized that KatG might be important for the epiphytic state of Xcc, prior to entry into the apoplast and colonization. To test this idea we analyzed the epiphytic fitness of the different Xcc strains on orange leaves, by monitoring the number of cells on the leaf surface over a period of 25 days. As shown in Fig 6B, at 5 days after inoculation the population size of the wild-type strain had increased about 100 times, whereas the number of *katG* mutant cells remained approximately constant. Then, the epiphytic populations of both strains declined almost at the same rate up to 20 days after inoculation. From that point forward, the number of wild-type cells was maintained but the mutant population continued to decrease, so that at 25 days post inoculation the difference in population size was more than 2 orders of magnitude. This result clearly indicates that KatG is required for the epiphytic persistence of Xcc on host leaves, which in term would determine the probability of disease occurrence.

The reduced epiphytic survival of *katG* deficient cells may be directly related to its impaired ability to form a structured biofilm (Fig 5), as biofilm growth is known to enhance bacterial resistance to adverse environmental conditions [61]. Epiphytic bacteria are usually exposed to a variety of environmental stresses, including desiccation, UV radiation, fluctuating temperatures and nutrient limitation; and the formation of matrix-enclosed biofilms is a common fitness strategy employed by epiphytic bacteria to survive and multiply on leaf surfaces [62]. Earlier studies have shown that Xcc forms complex biofilms on citrus leaves, being this important for epiphytic survival and disease development in plants [49]. Moreover, taking into account that KatG proved to be involved in the bacterial resistance to UV radiation (Fig 4), this catalase may also protect sunlight-exposed ephiphytic cells from oxidative damage produced by light-driven formation of ROS.

The cXcc*katG* strain exhibited an epiphytic survival pattern comparable to that of the *katG* mutant, even though it was able to complement the *in vitro* growth in SB medium (S1 Fig). In this regard, it is noteworthy that even though this strain was constructed by introducing *katG* gene under the control of its own promoter sequence on a low copy vector, the expression levels of *katG* are not necessarily identical to the normal expression levels in the wild-type strain, which contains only one copy of the gene in the chromosome. In fact, spectrophotometric determination of total catalase activity indicated that *katG* gene is somewhat ‘overexpressed’ in the complemented strain. The increased expression of *katG* may represent a stress for the bacterial cell given that all Xcc catalases require heme for proper folding and function. This stressful condition is probably more apparent when bacteria are in a minimal environment, such as the phyllosphere, than in the rich growth medium SB. Delmotte *et al*. have indicated that the phyllosphere constitutes a hostile environment for microorganism colonization because of the oligotrophic character of this habitat and the physical parameters that contribute to stress, such as UV radiation, temperature shifts, and the presence of reactive oxygen species [63]. Thus, the absence of complementation observed in the epiphytic survival assays may possibly be a consequence of the unfavorable condition of the c*katG* strain.

Requirement of catalases for successful colonization of host plants has been clearly demonstrated in different phytopathogenic species [35,38,64]. Indeed, in *P*. *syringae* pv. *tomato* DC3000 all three catalases (monofunctional KatB and KatE, and bifunctional KatG) are induced *in planta* and they each provide a degree of protection against ROS toxicity during pathogenesis. Furthermore, *katB* and *katG* single mutants both exhibit reduced growth *in planta* compared to the wild-type strain, indicating that these catalases play nonredundant roles in *P*. *syringae* virulence [35]. In agreement, our results collectively suggest that both monofunctional KatE and bifunctional KatG catalases contribute to Xcc virulence, although at different stages of the pathogenic process. KatG is required for the epiphytic survival of Xcc on host leaves prior to colonization, promoting virulence as the epiphytic population size of the pathogen determines the probability of disease occurrence [65]. Once inside the apoplast environment KatE induction provides protection against plant-produced hydrogen peroxide, thus favoring the successful establishment of Xcc in the host plant.

## Supporting Information

S1 Fig Growth curves of Xcc in SB medium. Xcc cultures were cultivated aerobically in SB medium at 28°C with shaking at 200 rpm. Aliquots were taken at the indicated times and measured for colony-forming capacity by serial dilution and plating on SB-agar. Colonies were counted after 48 h incubation at 28°C.
(TIF)
