# Supplementary figures and images for: KatG, the Bifunctional Catalase of Xanthomonas citri subsp. citri, Responds to Hydrogen Peroxide and Contributes to Epiphytic Survival on Citrus Leaves
Source: PLoS One. 2016 Mar 18;11(3):e0151657. doi: 10.1371/journal.pone.0151657 (PMC4807922; doi:10.1371/journal.pone.0151657)

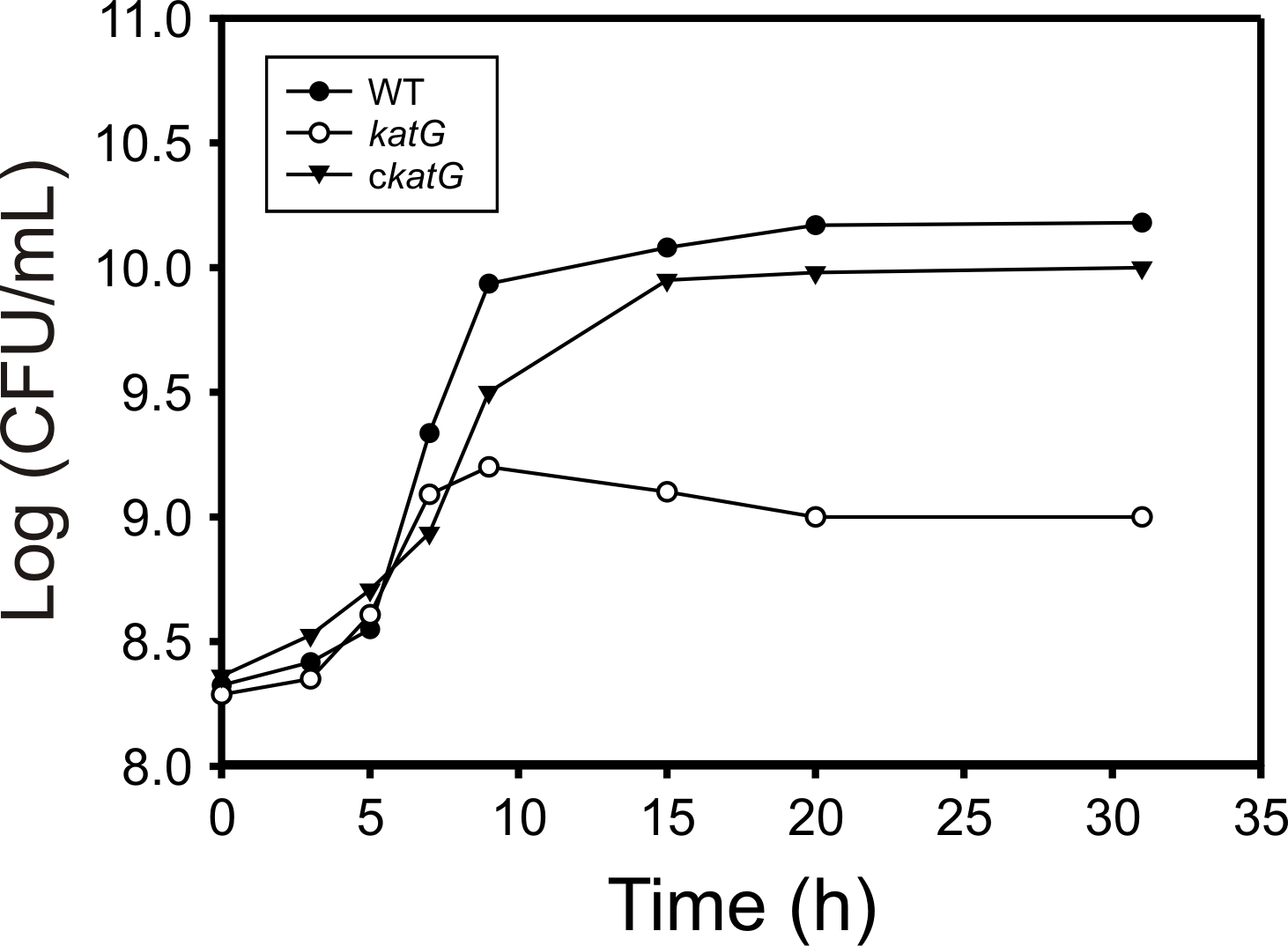

Supplement: S1 Fig — Xcc cultures were cultivated aerobically in SB medium at 28°C with shaking at 200 rpm. Aliquots were taken at the indicated times and measured for colony-forming capacity by serial dilution and plating on SB-agar. Colonies were counted after 48 h incubation at 28°C. (TIF) [file pone.0151657.s001.TIF]
